# COVID-19 Hijacking of the Host Epigenome: Mechanisms, Biomarkers and Long-Term Consequences

**DOI:** 10.3390/ijms262110372

**Published:** 2025-10-24

**Authors:** Alena D. Zolotarenko, Hakob M. Poghosyan, Victoria V. Sheptiy, Sergey A. Bruskin

**Affiliations:** Laboratory of Functional Genomics, Vavilov Institute of General Genetics, Russian Academy of Sciences, 119991 Moscow, Russia; pogosyan@vigg.ru (H.M.P.); sheptiy@vigg.ru (V.V.S.)

**Keywords:** COVID-19, epigenetics, long COVID, trained immunity, viral hijacking, epimarkers of disease severity

## Abstract

The epigenetics of COVID-19 is a rapidly expanding field that reveals how the SARS-CoV-2 virus initiates alterations in the host’s genome, influencing the susceptibility to infection, the disease severity, and long-term consequences, known as “long COVID.” In this review, we describe the mechanisms utilized by the virus to manipulate the host epigenome, suppressing antiviral responses and creating a favorable environment for viral replication. We also highlight virus-induced epigenetic changes across diverse cell populations that contribute to COVID-19 pathogenesis. Notably, the virus reprograms hematopoietic stem and progenitor cells, leading to long-lasting alterations in innate immunity, a phenomenon known as “trained immunity.” These epigenetic modifications are maintained in differentiated daughter cells and may explain the persistent inflammation and other symptoms of long COVID. Furthermore, we discuss emerging epigenetic biomarkers of disease severity, including methylation signatures in genes such as *AIM2*, *HLA-C*, and *PARP9*, as well as dysregulated miRNA profiles. Understanding this complex interplay between the virus and the host’s epigenetic landscape is crucial for developing new therapeutic approaches that target specific epigenetic modifications to suppress pathological processes and improve clinical outcomes for COVID-19 patients.

## 1. Introduction

### 1.1. Viral Invasion and Altered Pathways

The COVID-19 pandemic, triggered by the novel coronavirus SARS-CoV-2, emerged as one of the most devastating global health crises in modern history. First identified in Wuhan, China, in December 2019, the virus rapidly spread worldwide, leading to pandemic. As of September 2025, according to WHO data, confirmed cases surpassed 778 million globally, with approximately 7.1 million reported deaths, though true fatalities are estimated to range between 18.2 and 33.5 million due to underreporting and excess mortality [1].

The disease symptoms include fever or chills, dry cough, fatigue, shortness of breath or difficulty breathing, loss of taste or smell, muscle or body aches, nausea, vomiting, or diarrhea. The average duration of the symptomatic disease is about 10 days. The disease is highly transmissible and can be accompanied by pneumonia, systemic inflammation and multi-organ failure, especially in elderly groups and immunocompromised people. Furthermore, 10–30% of patients experience long COVID—chronic fatigue, brain fog, or cardiorespiratory issues for more than 3 months.

COVID-19 is caused by the SARS-CoV-2 virus, a positive-sense single-stranded RNA virus that initiates the invasion of a host cell by targeting the angiotensin-converting enzyme 2 (ACE2) receptor. This interaction is augmented by proteolytic cleavage mediated by the transmembrane serine protease 2 (TMPRSS2), which facilitates the viral entry or clathrin-dependent endocytosis [2,3]. Viral RNA release initiates ribosomal translation of replicase polyproteins pp1a/pp1ab, cleaved by viral proteases (M^pro^, PL^pro^) into functional subunits, including the RNA-dependent RNA polymerase (RdRp) complex [4]. Replication occurs within virus-induced double-membrane vesicles (DMVs) derived from the endoplasmic reticulum, where RdRp synthesizes nested subgenomic RNAs [5]. Viral proteins (e.g., Nsp1, ORF6) suppress interferon (IFN) responses by degrading host mRNA and blocking STAT1/IRF3 nuclear translocation [6].

In addition to blocking the interferon response, SARS-CoV-2 directly interferes with epigenetic regulation. The viral protein ORF8 plays a key role, mimicking histone H3, allowing it to bind to chromatin and suppress antiviral genes, including the components of the interferon pathway [7]. Simultaneously, other viral proteins induce ER stress and disrupt the autophagic flux [8,9]. These mechanisms collectively form a “one–two punch” that suppresses the primary recognition of the virus and the downstream IFN signaling, while shifting the immune response toward pro-inflammatory programs [6,7].

SARS-CoV-2 disrupts mitochondrial metabolism, increasing ROS production and inflammation, and activating cell apoptosis [10]. Viral Nsp4/Nsp6 induce ER stress, triggering the unfolded protein response (UPR) and eIF2α phosphorylation to inhibit host translation [8]. Epigenetic reprogramming via histone modification (H3K27me3) and DNA hypomethylation silences antiviral genes (e.g., *IFITM3*) while hyperactivating NF-κB and NLRP3 inflammasomes [7,11]. This drives gasdermin D-mediated pyroptosis and elevates cytokine production (IL-1β, IL-6) [12], causing tissue damage. Persistent infection and high ROS levels promote cellular senescence and non-coding RNA dysregulation (e.g., miR-155), contributing to long COVID [13].

### 1.2. Cell Populations Affected by the Disease

The initial cellular targets of SARS-CoV-2 are the epithelial cells expressing angiotensin-converting enzyme 2 (ACE2) receptors, particularly goblet cells in the nasal mucosa, type II pneumocytes in the alveoli, and enterocytes in the small intestine [14,15]. Single-cell RNA sequencing reveals that these cell types co-express ACE2 and the serine protease TMPRSS2, enabling efficient viral entry through S protein priming and membrane fusion [15,16]. Infection of alveolar type II pneumocytes is particularly important, as these cells maintain lung surfactant production and serve as progenitors for alveolar type I cells. Their dysfunction leads to alveolar collapse, impaired gas exchange, and aberrant repair mechanisms that promote pulmonary fibrosis [14,17]. Notably, *ACE2* itself was found to be an interferon-stimulated gene, potentially creating a feed-forward loop that enhances viral susceptibility in the abovementioned cell populations [15].

After the initiation of the infection, alveolar macrophages and neutrophils drive the pathological inflammation. Infected epithelial cells release DAMPs and PAMPs that activate alveolar macrophages, triggering NLRP3 inflammasome assembly and pyroptosis via gasdermin D cleavage, releasing IL-1β and IL-18. Concurrently, neutrophils infiltrate the lungs and undergo NETosis (neutrophil extracellular trap formation), releasing chromatin webs decorated with myeloperoxidase and citrullinated histones that propagate microthrombosis and endothelial damage [17,18]. This pro-inflammatory milieu is amplified by a delayed yet exaggerated type I interferon response, which fails to control early viral replication but later contributes to the development of the cytokine storm through STAT1/IRF3 signaling, further recruiting inflammatory monocytes and neutrophils to the lungs [15,18,19].

If the disease progresses to a severe form, lymphopenia occurs, characterized particularly by the depletion of CD8+ T-cells, as well as the impaired viral clearance [18,20]. SARS-CoV-2-specific T-cells exhibit exhausted phenotype markers (PD-1, TIM-3, LAG-3, TIGIT) and reduced cytotoxic activity, while dysregulated follicular helper T-cell (Tfh) responses compromise germinal center formation and neutralizing antibody production [18,19]. Molecular mimicry between viral epitopes (e.g., SARS-CoV-2 spike protein) and host antigens may provoke autoimmunity, with autoreactive T-cells targeting epithelial and endothelial cells, leading to enhanced tissue damage [17,19]. Additionally, persistent viral antigens presented by tissue-resident macrophages sustain IFN-γ and TNF-α production, driving a self-amplifying cycle of inflammation and tissue fibrosis that underlies post-acute consequences of the disease [14,17].

### 1.3. Cytokine Profiles and Long COVID

The pathophysiology of the systemic COVID-19 is characterized by a diverse array of immunomodulatory and pro-inflammatory cytokines, including interleukins IL-1β, IL-2, IL-6, IL-7, IL-10, IL-18, interferon-gamma (IFN-γ), tumor necrosis factor alpha (TNF-α), granulocyte–macrophage colony-stimulating factor (GM-CSF), interferon gamma-induced protein 10 (IP-10), macrophage inflammatory protein 1 alpha (MIP-1α), chemokine (C-C motif) ligand 2 (CCL2/MCP-1), along with D-Dimer and C-reactive protein (CRP). These cytokines are predominantly elevated in patients requiring intensive care unit (ICU) treatment, compared to those who are not critically ill. Such pathological hyperreaction of the immune system is named cytokine storm, reflecting a pathological hyperactivation of the immune response that disrupts communication between the infected cells and the host immune defense. High levels of pro-inflammatory cytokines contribute to the development of the acute respiratory distress syndrome (ARDS), which results in the critical manifestations of advanced COVID-19—widespread tissue damage and systemic organ failure, and could lead to COVID-19-related deaths [21,22].

**Figure 1:**
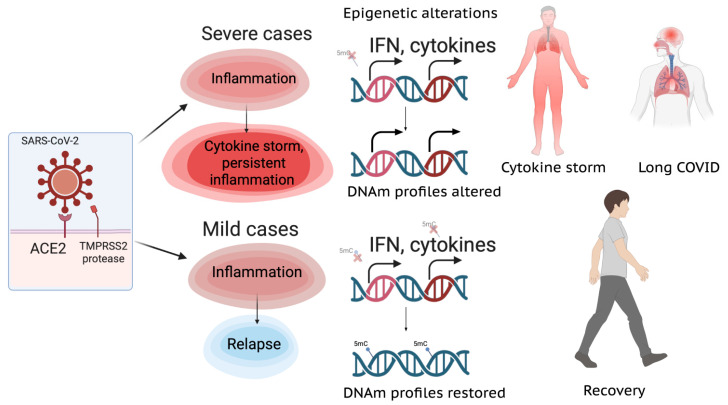
Epigenetic alterations in COVID-19 lead to severe cases and long COVID. The illustration was created at BioRender.com.

**Figure 2:**
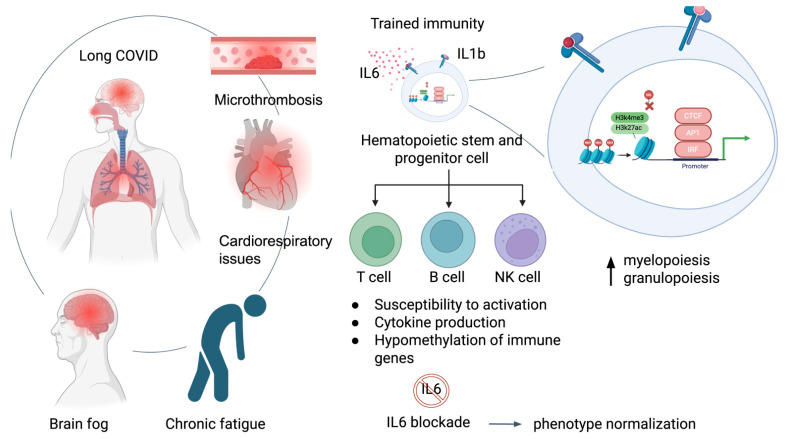
Long COVID symptoms are associated with the phenomenon of trained immunity. The illustration was created at BioRender.com.

Even after the disease resolution, a significant percentage of the patients still suffer from the phenomenon named long COVID. It is currently accepted that chronic low-grade inflammation is present in systemic circulation even after the disease resolution, affecting different organs—leading to dysregulated activity of brain microglia that releases cytokines and produces neuroinflammation and “brain fog”, to the development of immunothrombosis of blood vessels and organs, linked to chronic inflammation with microclot formation, decreased tissue perfusion and ischemia as well as myalgic encephalomyelitis/chronic fatigue syndrome (ME/CFS) and dysautonomia occurring in some patients, that could involve development of autoimmunity [23]. Studies have identified epigenetic mechanisms to contribute to the development of long COVID-19 (Figure 1 and Figure 2) [24,25]. The acute cytokine storm in severe COVID-19, dominated by the elevated IL-6 and to a lesser extent IL-1b profiles, epigenetically reprograms hematopoietic stem and progenitor cells (HSPCs) via altered chromatin accessibility and transcriptional factors, establishing trained immunity as the bridge to long COVID symptoms [26]. This imprint persists in circulating monocytes, maintaining open inflammatory enhancers, hyperresponsiveness to stimuli, and low-grade systemic inflammation that sustains long COVID manifestations such as fatigue, cognitive dysfunction and microthrombosis. IL-6 blockade partially reverses such epigenetic alterations in monocytes [24,26].

## 2. Epigenetics of COVID-19

In general, epigenetic alterations could be triggered by various factors, including host immune response, psychological stress, preexisting health conditions, oxidative stress, behavioral and environmental factors, as well as advancing age. Furthermore, the viral infection itself may influence the host’s immune responses related to the inflammation by altering epigenetic mechanisms, which can play a critical role in determining the severity of the disease and the risk of mortality. It was demonstrated previously that coronaviral infection is linked to the accelerated aging of the host immune system via epigenetic mechanisms such as DNA methylation and transcriptional suppression, disrupting the pathways of antigen presentation and the expression of major histocompatibility complexes [27].

Epigenetic reprogramming plays a significant role in the pathogenesis and immune response to COVID-19. The infection triggers global DNA methylation changes, histone modifications, nucleosome positioning and chromatin remodeling, which can affect the host’s immune response and viral replication efficiency. These epigenetic changes can drive differences in infection susceptibility and disease severity based on tissue type, biological age, and sex. The epigenetic profiles evaluated by blood analyses could represent altered blood cell populations associated with a particular stage of the disease. Persistent epigenetic alterations in hematopoietic stem cells and immune cells can lead to prolonged inflammatory responses and altered immune function. Understanding these mechanisms opens up potential therapeutic avenues, such as targeting specific epigenetic modifications to reduce the disease severity and improve patient outcomes [25].

### 2.1. Epigenetic Alterations of Different Cell Populations

The recently identified methylation patterns specific to different cell types allow tracing of the contributions of different cell populations to the disease progression and the death dynamics at the cellular level in health and disease.

Recent evidence reveals extensive epigenetic alterations in monocytes in COVID-19, including the increased chromatin accessibility at genes related to cytokine production and leukocyte activation, even during the recovery phase. These alterations likely arise from the reprogramming of upstream hematopoietic stem and progenitor cells (HSPCs) and represent the trained immunity (Figure 2) [24].

An analysis of isolated circulating hematopoietic stem and progenitor cells (HSPC) from peripheral blood, combining single-cell RNA/ATAC-seq, captured the diversity of circulating HSPC as seen in bone marrow, allowing for the non-invasive investigation of hematopoiesis as well as epigenomic changes after COVID-19. In cases of severe COVID-19, both transcriptomic and epigenetic changes in HSPC and its progeny innate immune cells were observed, and they persisted for months to a year. Inflammatory pathways as well as transcription factors (including IRF, AP-1, and CTCF) were activated, HSPCs displayed long-lasting increases in myelopoiesis and granulopoiesis, and circulating monocytes responded more strongly to stimulation. The epigenomic changes in HSPC were retained and passed onto differentiated progeny innate immune cells, illustrating the concept of trained immunity. Trained immunity is defined as immunological memory of the innate immune system maintained by epigenetic modifications and capable of protection against secondary pathogen challenge, but for COVID-19 [28].

However, it was demonstrated that administration of an anti-IL-6 blockade during acute severe COVID-19 could normalize the persistent phenotypes [26]. IL-6 appears pivotal for imprinting durable epigenetic modifications in monocytes during acute infection, with IL-1β playing a contributory role [24].

A recent study using scATAC-seq and Ti-ATAC-seq (a method combining FACS with single-cell T cell-receptor sequencing and ATAC-seq) has shown the establishment of trained immunity in a newly described COVID-associated population of T-bet-enriched CD16+ and IRF1-enriched CD14+ monocytes. The analysis revealed alterations in chromatin state for almost all the analyzed immune cell types of the convalescent COVID-19 individuals. Epigenomic analysis showed a clear progression trend in CD14+ and CD16+ monocytes, moving from homeostatic state to mature inflammatory effector phenotypes through trained and activated states. In convalescent individuals, the B-cell lineage demonstrated a rapid maturation trajectory from immature B cells to plasma cells, and the regulation of the AP1 transcription factor was activated. The naïve B-cells of the convalescent individuals showed activity of SPI1, EBF1, IRF4, and POU2F2, the transcription factors vital for B-cell survival, differentiation, and receptor signaling. Ti-ATAC-seq indicated a clonal expansion of putative SARS-CoV-2-specific effector and memory CD8+ T cells characterized by the epigenomic signatures. The observed maturation of different populations of immune cells may be the consequence of the elevated levels of IFNγ in the serum of COVID-19 patients. High levels of IFNγ lead to the activation of TBET, which could act as the inductor of monocyte activation and maturation, IgG+ memory B cell development and effector and memory T cell differentiation [29].

Spector and colleagues [30] used whole-genome bisulfite sequencing (WGBS) to analyze nasal samples from COVID-19 patients with mild and severe symptoms. High levels of differential DNA methylation in intergenic regions and low methylated regions (LMRs) demonstrated the importance of distal regulatory elements in gene regulation in COVID-19. Differential methylation was observed for pathways implicated in immune cell recruitment and function, and inflammatory response (e.g., impaired neutrophil adhesion in severe disease because of *FUT4* hypermethylation, or hypermethylation of *ELF5* binding sites leading to downregulation of ELF5 targets in the nasal cavity). Observed hypermethylation of enhancer-like elements in myeloid and lymphoid cells suggested disrupted immune cell recruitment and function [30].

Analysis of methylation profiles combined with single-cell transcriptomes of nasal cells of COVID-19 patients showed that hypermethylation of ciliary function genes (e.g., *FOXJ1*, *DNAH5*) led to sustained transcriptional repression up to 12 months post-infection, while hypomethylation in immune genes (e.g., chemokine receptors) upregulated inflammatory pathways in monocyte-derived macrophages [31]. In addition, symptom-dependent alterations were observed: patients with persistent respiratory post-COVID symptoms showed stronger repression of ciliary genes.

A study on single-cell multi-omics analysis of transcripts and proteins of NK lymphocytes in COVID-19 patients was performed to characterize the innate immunological response to infection as well as vaccination against COVID-19. Vaccinated patients showed upregulated activation markers (IFN-γ, GZMB, NKG2D) and cytokine signaling pathways (IL-2/STAT5) while convalescent patients had elevated exhaustion markers (PDCD1, LAG3) and reduced cytotoxicity compared to vaccinated individuals. JAK-STAT and NF-κB pathways were hyperactivated in vaccinated groups. TGF-β signaling was heightened in convalescent patients, associated with suppressed NK function. These findings highlight NK cells as therapeutic targets for enhancing immune response in COVID-19 [32].

### 2.2. Epigenetic Modifications of the Host Genome Induced by the Virus

Recent studies have shown that coronaviruses cause specific epigenetic changes in the host cells to create a conducive microenvironment for replicating, assembling, and spreading [7,33,34]. Epigenetic regulation during virus–host interactions involves DNA methylation, histone modification, and microRNA modification, influencing viral life cycles, gene expression, and latency. This interplay affects both virus persistence and host cell responses, offering insights for potential novel treatments.

SARS-CoV-2 infection leads to significant changes in the host’s epigenetic landscape. Infected cells trigger various signaling pathways through epigenetic modifications to create an antiviral environment. Simultaneously, the virus attempts to suppress essential genes, altering transcriptional programs that inhibit antiviral responses and promoting its own survival and replication [34].

**Figure 3:**
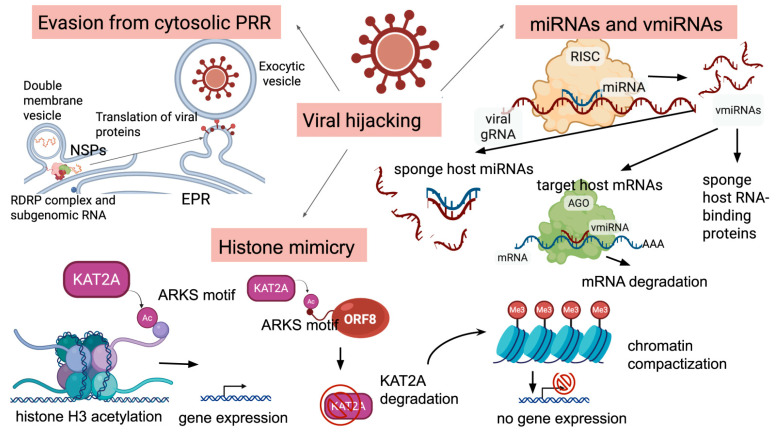
SARS-CoV-2 hijacking strategies. The illustration was created at BioRender.com.

There are several approaches utilized by the virus to alter host epigenetic profiles. One of the ways SARS-CoV-2 influences host epigenetic regulation is via the ORF8 protein, which functions as a histone H3 mimic. ORF8 contains an “ARKSAP” motif (amino acids 50–55) identical to key regulatory regions of the histone H3 N-terminal tail (Figure 3). This motif binds chromatin and interacts with epigenetic regulators, including lamins, histone H3, and histone acetyltransferase KAT2A. Acetylation of lysine in ORF8’s ARKSAP motif mimics H3K9ac/H3K27ac modifications, disrupting chromatin remodeling and suppressing transcription of antiviral genes. Deletion of the ARKSAP motif reduces chromatin compaction and viral replication capacity, confirming ORF8’s role in epigenetic sabotage [7]. This histone mimicry is a direct tool for immune evasion, blunting the cell’s intrinsic defense system and facilitating viral replication, thereby increasing the host’s susceptibility to severe infection. However, the role of ORF8 as a histone mimic is a subject of ongoing scientific debate, highlighting the complexity of viral epigenetics. While some studies, such as that by Kee et al., provide compelling evidence for the KAT2A degradation mechanism, other research has failed to confirm that ORF8 functions in this capacity. Research by Liu and colleagues pointed out that ORF8 is an ER lumen protein but not a nuclear one; it develops mixed disulfide complexes with ER-resident oxidoreductases, which activate the unfolded protein response, leading to the remodeling of the ER to facilitate viral replication. In addition, they noted that histones are among the most abundant proteins in an eukaryotic cell, so there should be a very high presence of the ORF8 protein in the nucleus to make a significant contribution to the levels of free epigenetic regulators [35]. Despite this controversy over the precise mechanism, the functional outcome is consistent with a broader viral strategy of epigenetic disruption. This multifaceted attack on the host epigenome, whether through confirmed histone mimicry as seen in influenza NS1 or the proposed but contested mechanism of ORF8, is a cornerstone of SARS-CoV-2’s ability to cause severe disease by systematically silencing the immune response.

Another epigenetic route altered by SARS-CoV-2 is the upregulation of DNA methyltransferases (DNMT1/DNMT3A), causing hypermethylation of immune response gene promoters (e.g., interferon genes), which blocks viral recognition by TLR and RIG-I receptors [33,34].

One more strategy, realized by the virus during infection, is altering chromatin accessibility and nucleosome positioning in the host cells to favor viral replication. In general, nucleosome positioning along the DNA helix determines the accessibility of DNA to the regulatory machinery during transcription or DNA replication. Actively transcribed chromatin is nucleosome-depleted, allowing RNA polymerase and transcription factors to bind effectively. Research has identified alterations of chromatin accessibility of immune genes in PBMCs of COVID-19 patients, with the most extensive remodeling occurring in CD14+ monocytes—while in mild cases classic antiviral pathways in this cell type are upregulated, in more severe cases they are diminished, leading to dysregulated immune responses [29,36]. A research evaluating nucleosome positioning in cells infected with different human coronaviruses revealed that nucleocapsid proteins of all the coronaviruses analyzed induced alterations in nucleosome positioning and availability of gene promoters to transcriptional machinery [37]. SARS-CoV-2 nucleocapsid protein induced alterations at nucleosome positioning and chromatin accessibility at genes involved in innate immune response, tight junction interactions, coagulation and hormone signaling—all the pathways linked to severe COVID-19 outcomes and post-acute sequelae. Promotors of genes linked to viral myocarditis and olfactory transduction shifted from a silent to an active state, suggesting a link between nucleosome positioning alterations and the disease complications [37].

SARS-CoV-2 hijacks host epigenetic mechanisms, altering gene expression and immune responses, thereby facilitating viral entry, replication, and immune evasion through modifications like DNA methylation and histone alterations [38]. The virus enters the host cell after successful binding and cleavage of the host’s angiotensin-converting enzyme 2 (ACE2) receptors, widely expressed in different tissues of the human body. The expression of ACE2 in the upper respiratory tract, lungs and olfactory epithelium is rather low, while in the small intestine, heart, kidney and testis it is higher than in other tissues [39]. *ACE2* transcription is activated by transcription factors including Ikaros, HNFs, GATA6, STAT3 or SIRT1, and reduced by the transcription factor Brg1-FoxM1 complex or ERRα. *ACE2* levels are also regulated by histone modification or miRNA-induced destabilization, and the protein levels and stability are regulated by phosphorylation, ubiquitination, SUMOylation, methylation and lysosomal degradation [40]. The tissue/cell type expression of ACE2 is regulated by epigenetic modifications [39].

Research indicates a significant sexual dimorphism in *ACE2* promoter methylation, which contributes to differential COVID-19 susceptibility and severity between men and women. Under normal conditions, men exhibit lower levels of *ACE2* gene methylation in lung cells compared to women. This hypomethylation is associated with higher baseline *ACE2* expression, which, while potentially mitigating severe lung injury by regulating angiotensin levels, also facilitates greater viral entry and results in a higher viral load upon infection. In contrast, women generally have higher promoter methylation, which would typically suppress *ACE2* expression. However, this is counteracted by hormonal and genetic factors; estrogen can upregulate *ACE2* expression. In addition, the *ACE2* gene is localized on the X chromosome, Xp22.2, an area where genes could escape X-inactivation, leading to its potential overexpression in females [41,42]. This combination creates a protective paradigm for women: the hormonal and genetic predisposition for higher ACE2 availability may help buffer the dysregulation of the renin–angiotensin system (RAS) during infection, preventing the progression to a more severe disease form.

The relationship between *ACE2* methylation and disease outcome is complex and involves a delicate balance. On one hand, lower *ACE2* expression (as often associated with higher promoter methylation) can be protective against initial infection by reducing the number of available viral receptors. On the other hand, once infected, a higher level of *ACE2* expression may be beneficial in preventing severe disease by maintaining the enzymatic activity that degrades angiotensin II. This duality is reflected in the conflicting data on specific methylation sites, such as cg21598868, which shows greater variability in women [43]. *ACE2* methylation could differ not only between sexes but also between different tissues: research by Andres Cardenas and colleagues describes CpGs differentially methylated by sex, with 12 sites having lower DNAm (mean = 12.71%) and 3 sites greater DNAm (mean = 1.45%) among females relative to males. Such variability suggests a molecular link for gender-specific responses to COVID-19, where the epigenetic regulation of ACE2 acts as a key modifier, influencing both the initial susceptibility to the virus and the subsequent pathological course of the disease [44]. In addition, DNA methylation analysis of airway epithelium showed age-dependent methylation decline of cg085599149 adjacent to the *ACE2* transcription start point [45].

SARS-CoV-2 causes alteration of the *ACE2* through the methylation at three different sites. In COVID-19 patients, generally, *ACE1* expression is suppressed while *ACE2* is always upregulated, enhancing host cell susceptibility to virus infection [46]. Moreover, soluble ACE2 protein and exosomal ACE2 protein facilitate SARS-CoV-2 infection into host cells [40].

A study by Taefehshokr and colleagues demonstrated that SARS-CoV-2 interferes with antigen presentation by downregulating major histocompatibility complex (MHC) II on antigen-presenting cells. The viral main protease, NSP5, suppresses the expression of the MHC II regulatory protein CIITA across a range of professional and non-professional APCs, thereby limiting MHC II expression. This suppression occurs via a pathway in which NSP5 delivers HDAC2 to the CIITA promoter via interactions between NSP5 and promoter-bound IRF3. HDAC2 then deacetylates histones at the CIITA promoter, decreasing CIITA expression, and thereby blocking MHC II expression [47].

Coronaviruses utilize NSP3, NSP4, and NSP6 to hijack the endoplasmic reticulum membrane to form double-membrane vesicles, where the virus replicates to evade cytosolic pattern-recognition receptor sensing (Figure 3) [48]. SARS-CoV-2 NSP1 was shown to cause depletion of antiviral factors Tyk2 and STAT2, which may be due to the NSP1 or NSP14-mediated inhibition of the overall cell translation [49,50]. Moreover, SARS-CoV-2 NSP14 was shown to disturb the interferon signaling by IFNAR1, targeting lysosomal degradation with a consequent decrease in STAT1 phosphorylation. In addition, SARS-CoV-2 E, ORF3a, and ORF7a block autophagic turnover, whereas M may differently interact with the autophagic machinery and Nsp15 inhibits de novo autophagy induction. In the viral context, autophagic flux is blocked [9].

Protein–protein interaction analysis of the SARS-CoV-2 interactome allowed researchers to identify host proteins modulating SARS-CoV-2 infection in human cells [51]. Inhibition of the epigenetic regulators bromodomain-containing protein 4 (BRD4) and histone deacetylase 2 (HDAC2), along with ubiquitin-specific peptidase (USP10), enhanced SARS-CoV-2 infection, meaning that the abovementioned epigenetic regulators are important regulators inhibiting the viral spreading. Earlier, it was shown that HDAC2 can act as either a restriction or dependency factor for various viruses. For instance, HDAC2 acts as a dependency factor for herpes simplex viruses (HSV), and inhibition of HDAC2 reduces human adenovirus expression and replication. On the other hand, HDAC2 contributes to the antiviral response against influenza viruses [51].

### 2.3. Altered Host miRNome

The systemic response to SARS-CoV-2 infection is characterized by a significant alteration in the host’s circulating miRNA profile. Studies analyzing circulating miRNAs from serum and plasma have predominantly observed a widespread downregulation of host miRNA expression, particularly in patients with severe symptoms. However, the cell machinery for miRNA production—such as AGO2, DICER1, DGCR8, DROSHA, and XPO5—revealed no significant downregulation in patients with severe COVID-19 compared to those with non-severe disease or uninfected individuals, indicating that the observed decrease in miRNA levels is not due to a systemic failure in the core machinery responsible for miRNA production. Instead, it points toward more targeted mechanisms, such as the potential sequestration or selective degradation of host miRNAs by the virus or by host-induced pathological feedback loops [52].

The interaction between miRNAs and viruses is dynamic: miRNAs can directly bind to viral RNA sequences to influence the infection cycle, leading to the degradation of the viral RNA or the suppression of its translation into proteins, effectively inhibiting viral replication. For example, miR-122 has known functions in the HCV life cycle, acting as an RNA chaperone (or “riboswitch”) to help form the viral internal ribosomal entry site (IRES), providing genome stability, enhancing viral translation and participating in virion assembly [53]. Consequent research has shown that human miR-122 also binds the SARS-CoV-2 RNA genome with high affinity, suggesting the perspective of repurposing anti-HCV RNA-based drugs, such as Miravirsen, to treat COVID-19 [54].

Moreover, miRNAs can also affect virus spread by modulating host cell factors required for viral assembly and release. miRNAs can target cellular genes involved in these processes, thereby impairing the virus’s ability to spread within the host. For example, miR-29a targets the 3′ UTR of the HIV-1 genome, leading to decreased viral replication and spread, suggesting a reverse correlation between the miRNA and HIV-1 replication [55]. Hsa-miR-29a-3p is significantly downregulated in severe COVID-19 and recent in silico data showed that it has a high affinity to the SARS-CoV-2 genome and may be an attractive therapeutic target. Furthermore, it was predicted to bind the 3′-untranslated regions of both IL17RA and THBS1 mRNAs, implicating a role of the virus-induced downregulation of this miRNA in the COVID-associated cytokine dysregulation [56].

Besides the general downregulation of miRNome, certain miRNA expression patterns were found to correlate with the clinical trajectory of COVID-19; some of them are upregulated in mild or protective responses, while others are associated with severe disease and poor outcomes. To name just a few, miR-27a-5p (targeting TGF–beta pathway) was found to be overexpressed in subjects with mild COVID-19, suggesting a potential protective role against the progression to a severe form of the disease. TGF-β1 is a master regulator of immune reaction and pulmonary fibrosis in COVID-19 patients. TGF-β1 induces chronic immune reaction by regulating antibody switching from IgG to IgA and this correlates with prolonged ICU stays of more than seven days [57].

Conversely, the upregulation of miR-155-5p or miR-21-5p (miRNAs regulating cytokine signaling and inflammatory response), was consistently reported and is strongly linked to disease severity and a worse prognosis. On the other hand, several miRNA families were found to be downregulated; for example, the let-7 family was consistently observed to be downregulated in COVID-19 patients, and the downregulation facilitated viral entry and cytokine storm. Other miRNAs like hsa-miR-182, hsa-miR-183, hsa-miR-205, and hsa-miR-2110 also exhibited significant downregulation in response to the infection [58,59,60].

The altered miRNA profile could serve as a promising tool for the prediction of the severity of COVID-19, reflecting the clinical symptoms of the infection, such as the need for oxygen therapy and concomitant pneumonia. For example, evaluation of the levels of hsa-miR-150–5p, hsa-miR-423, hsa-miR-370, hsa-miR-369–3p, miR-144–3p, and miR-144–5p could be used to distinguish severe and non-severe COVID [61,62], and low expression of miRNAs miR106a-5p, miR17-5p, miR181a-5p, miR191-5p, miR20a-5p and miR451a, especially in the initial phase of the disease, is associated with an unfavorable clinical course of SARS-CoV-2 infection (admission to the ICU) [63].

The influence of miRNA dysregulation extends beyond the primary sites of infection to contribute to the multi-organ complications characteristic of severe COVID-19. For instance, the dysregulation of miR-155 is not limited to inflammatory pathways. It also represses the AGTR1 gene, which codes for the angiotensin II type 1 receptor (AT1R), a central component of the renin–angiotensin–aldosterone system (RAAS). The RAAS is a primary regulator of blood pressure, fluid balance, and cardiovascular function. By modulating this system, the dysregulation of miR-155 can mechanistically bridge the gap between a respiratory viral infection and systemic complications such as cardiovascular disease, thrombosis, and hypertension, providing a deeper understanding of the multi-systemic nature of the disease [64,65,66].

### 2.4. Viral miRNA-like RNAs

The host’s miRNA-mediated defenses are met with a sophisticated counter-strategy from the virus itself. SARS-CoV-2 has been shown to produce its own small, viral miRNA-like RNAs (v-miRNAs) that hijack the host’s cellular machinery to modulate gene expression for its own benefit (Figure 3). A key example is CoV2-miR-O7a, a small RNA derived from the viral ORF7a region [60]. This v-miRNA is processed by the host’s Dicer enzyme and associates with Argonaute proteins, which are core components of the RNA interference pathway. The presence of CoV2-miR-O7a has been confirmed in infected cells and in patient nasopharyngeal swabs, demonstrating its active role during infection. Authors identified the putative targets of CoV2-miR-O7a, including BATF2, a transcription regulator of interferon signaling, and HSPG2, a core protein of a large multidomain proteoglycan participating in the attachment of some viruses, including coronavirus NL63 [67].

Another viral miRNA, CoV2-miR-O8, was found to be highly represented in nasopharyngeal samples from patients with COVID-19 infection, to have features consistent with Dicer and Drosha generation, as well as interaction with Argonaute, and to target human mRNAs and microRNAs [68].

Several candidate miRNAs encoded in the SARS-CoV-2 genome were predicted bioinformatically as well as their putative targets in the human genome. Mir147-3p was predicted to suppress the expression of human EXOC7 (involved in exocytosis), RAD9A (involved in DNA repair), and TFE3 (a transcription factor). This can affect immune response processes and cytoskeleton organization in infected cells [69].

Another research identified SARS-CoV-2-encoded SCV2-miR-ORF1ab-1-3p and SCV2-miR-ORF1ab-2-5p, targeting several genes in the type I interferon signaling pathway—IRF7, IRF9, STAT2, OAS1, and OAS2. The results suggest that SARS-CoV-2 uses its miRNAs to evade the type I interferon response and links the functional viral sequence to the susceptible genetic background of the host [70].

Besides viral miRNA-like RNAs, the virus hijacks host miRNAs to modulate host biological processes. For instance, the virus can cause downregulation of specific host miRNAs that normally act as suppressors of viral replication, thereby relieving this natural inhibition. The 3′UTR of the viral spike (S) gene acts as a sponge and downregulates host miRNAs such as miR-296 and miR-602 to disturb cell growth and cytokine signaling (e.g., IL-6 and TNF-α) [71]. Conversely, it can upregulate other host miRNAs that promote a pro-viral state, such as miR-21-3p, upregulated upon invasion and targeting immune genes, facilitating viral immune evasion [72], or miR-146a and miR-155, dysregulated to suppress interferon and NF-κB pathways, reducing antiviral responses [73]. Such sophisticated manipulations of the host’s post-transcriptional regulatory network allow SARS-CoV-2 to modulate fundamental biological processes to enhance its own survival and pathogenicity.

### 2.5. Accelerated Epigenetic Aging Associated with COVID-19

The investigation into epigenetic patterns following COVID-19 infection reveals significant insights into accelerated biological aging and enhanced epigenetic drift. Studies indicate that the epigenetic signature, already present at the time of hospital admission, can significantly predict risk of severe outcomes [74]. Six months post-infection, patients still exhibit notable alterations of DNA methylation, suggesting a potential link to long-COVID symptoms such as fatigue and neurological issues [75]. It was shown, using the EPIC methylation array, that COVID-19 patients, especially those with more severe COVID-19, show increased epigenetic age and telomere shortening compared to healthy individuals. Accelerated epigenetic aging (EAA) is linked to a higher risk of severe COVID-19 and post-COVID-19 syndrome; however, longitudinal DNA methylation profiling analysis found that the accumulation of epigenetic aging from COVID-19 syndrome could be partly reversed at late clinic phases in some patients [76]. Another study confirmed significant accelerated epigenetic aging but no acceleration of telomere shortening in severe COVID-19 cases [77]. Notably, deceased patients exhibited higher EAA compared to those who recovered, as measured by the Horvath and PhenoAge clocks. Telomere attrition acceleration was also observed, and there was a significant change in the dynamics of telomere attrition acceleration when comparing patients who recovered versus those who died. This suggests that EAA may be a critical factor in determining patient outcomes and indicates a potential link between telomere dynamics and mortality [77]. While the majority of research supports the notion of accelerated biological aging post-COVID-19, some studies challenge this view. For example, a research by Franzen and colleagues highlights the alterations in the immune cell populations of COVID-19 patients compared to the datasets used for the development of different epigenetic clocks, which could explain the absence of EAA in their research and indicate a need for further investigation into the complexities of epigenetic responses to viral infections [78].

The potential mechanisms driving age acceleration are complex and multifactorial. The excessive cytokine and ROS production and chronic inflammatory background is a well-known drivers of epigenetic alterations and cellular senescence. Furthermore, the SARS-CoV-2 virus itself directly interferes with host epigenetic machinery, leading to alterations of the epigenetic profiles, shifting cells to a more senescent state.

#### Enhanced Epigenetic Drift

The concept of enhanced epigenetic drift following COVID-19 infection has garnered attention due to its potential implications for long-term health outcomes. It was shown that epigenetic alterations, especially altered DNA methylation, may play important roles in the persistence of symptoms associated with long COVID and influence the disease severity [74]. Epigenome-wide studies show significant differences in DNA methylation between severe and mild cases, namely in the pathways of interferon signaling and viral response, suggesting that these changes can predict clinical outcomes [79].

There is controversial data on the roles of DNA methyltransferases in COVID-19. A study comparing mild, moderate, severe and critical disease states identified inhibited expression of DNA methyltransferases DNMT1 and DNMT3A in COVID-19 patients, and alterations in promoter methylation of immune response genes. *TLR4* and *TNF-α* had increased promoter methylation, and the methylation of the *TNF-α* promoter increased as disease severity increased. Significantly less methylation of the *TLR3* promoter was observed in patients with a positive outcome (recovery). Critically ill patients had lower *HDAC3* expression levels. In milder cases, the global 5-mC levels were lower than those in more severe cases [80].

Understanding the mechanisms and implications of epigenetic drift may help to develop strategies for monitoring and managing long-term health effects post-infection. However, while enhanced epigenetic drift presents significant concerns, not all individuals experience severe long-term effects, indicating variability in epigenetic responses to COVID-19. This variability underscores the need for further research to understand the underlying mechanisms and potential for recovery.

### 2.6. Epimarkers of Disease Severity and Outcome

Recent studies have identified specific CpG sites that can accurately differentiate COVID-19 patients from other disease groups, achieving up to 100% predictive accuracy (Table 1). In addition, the identification of reliable DNA methylation markers opens avenues for targeted therapies, as certain genes may serve as druggable targets.

The choice of a DNA methylation analysis technology platform is a trade-off between genome coverage, resolution, and cost. This directly affects the consistency of the results obtained in different studies. Genome-wide bisulfite sequencing (WGBS) is considered the “gold standard” as it provides almost complete genome coverage and a resolution of up to a single nucleotide, making it ideal for exploratory research. However, this technique involves bisulfite treatment, which leads to DNA degradation and requires significant computational resources for analysis, limiting the use of this approach in larger clinical studies. In contrast, Illumina Infinium Methylation EPIC microarrays offer a cost-effective and efficient solution for epidemiological studies, demonstrating high consistency in the results obtained. A key limitation of microarrays for analyzing DNA methylation is their fixed design, which only covers about 3% of CpG sites in the genome, mostly in promoter regions. This limits the ability to detect new markers in distal regulatory elements [81].

Methods for analyzing DNA methylation of individual cells, such as SCBs-seq, are crucial for studying COVID-19. These methods allow access to the heterogeneity of cell populations participating in the immune response reactions and to analyze the alterations at the level of individual cells. Thus, it becomes possible to differentiate true alterations in methylation levels from changes in cellular composition, which is a major challenge when analyzing whole blood samples [82,83].

Third-generation sequencing technologies like Oxford Nanopore allow direct detection of modifications on native DNA molecules. This eliminates conversion-related artifacts and allows the analysis of methylation in complex genomic regions due to long read lengths. The accuracy of determining the methylation status of individual sites in nanopore sequencing is slightly lower compared to full-genome bisulfite sequencing (WGBS). However, the total methylome profiles obtained using ONT technology are in good agreement with WGBS data, with a coefficient of determination of around 0.95 [84,85]. Another advantage of this technology is its potential suitability for epigenetic analysis at the level of individual cells [86].

A study, drawing from 865,859 methylation sites, discovered two miniature sets of Infinium MethylationEPIC sites, each having eight CpG sites to interact with each other and disease subtypes. They led to the nearly perfect (96.87–100% accuracy) stratification of COVID-19 patients from patients with other diseases or healthy controls. Such level of accuracy suggests that these biomarkers could be crucial for early diagnosis and differentiation of COVID-19, and can jointly explain some post-COVID-19-related conditions. These CpG sites and the optimally performing genomic biomarkers reported in the literature become potential druggable targets. Among these CpG sites were cg16785077 (gene *MX1*), cg25932713 (gene *PARP9*), and cg22930808 (gene *PARP9*) [87].

A study by Castro de Moura et al. analyzed the DNA methylation status of 850,000 CpG sites in peripheral blood samples from 407 confirmed COVID-19 patients and identified a specific DNA methylation signature, termed “EPICOVID,” that is associated with the severity of the disease. The study included patients with mild and severe clinical courses (requiring respiratory support) and found that the DNA methylation status of 44 CpG sites was associated with clinical severity. Of these loci, 23 were located in 20 promoters of annotated coding genes, including the inflammasome component *AIM2* and the *HLA-C*. These sites could act as epigenetic susceptibility loci for respiratory failure in COVID-19 patients [88].

*HLA-C* plays a critical role in adaptive immunity by presenting viral peptides to cytotoxic T-cells. Simultaneously, it is a key regulator of innate immunity, interacting with NK cell receptors to distinguish healthy cells from the infected ones. A separate study focused specifically on *HLA-C* methylation in COVID-19 [89]. Analysis of 470 peripheral blood samples found it was significantly lower in COVID-19 patients with moderate, severe, and critical disease compared to controls. The study also noted significant gender-related differences, with men showing higher methylation levels in all disease stages compared to women, and the highest methylation percentage was observed in the severe group of male patients.

In a study comparing severe and mild cases in an Italian cohort, researchers identified a list of 21 CpG sites, an epigenetic signature, that could be used to distinguish severe and mild cases. It had a significant association with severity, with an odds ratio (OR) of 2.55, and had more predictive power of severe outcomes than models based on clinical factors alone. Genes such as

*MAPK10* and *PIK3CD* were found to be hyper-methylated in patients with a poor prognosis, while genes including *ARID5A*, *CD226*, *CD244*, *IL1R1*, and *STAT6* were annotated from hypo-methylated CpG sites in the same patient group [74].

Konigsberg and colleagues have performed an epigenome-wide association study (EWAS) utilizing a customized high-resolution MethylationEPIC array to analyze the peripheral blood from a cohort of 164 COVID-19 patients and 296 controls. This investigation identified a robust, COVID-19-specific epigenetic signature. Genome-wide analysis identified 13,033 significantly differentially methylated sites, with a notable enrichment in genes related to interferon signaling and viral response. Predictive models utilizing sparse regression machine learning demonstrated high efficacy, achieving cross-validated AUC values of 79.1% (for hospitalization), 80.8% (for ICU admission), and 84.4% (for mortality) [79].

A study [90] aimed to evaluate the implications of DNA methylation in COVID-19 progression by means of a genome-wide DNA methylation analysis combined with DNA genotyping of 473 SARS-CoV-2 positive and 101 negative individuals. The results revealed the existence of epigenomic regulation of functional pathways associated with COVID-19 progression and mediated by genetic loci. Sites, hyper-methylated in severe disease, were enriched in genes regulating AKT signaling (e.g., PIP3), while hypomethylation affected interferon-inducible genes (e.g., *OAS1*, *PARP9-DTX3L*, *IFIT3*) and lymphocyte activation genes (e.g., *CD38*, *LAT*). These epigenetic changes were linked to increased neutrophil counts and disrupted immune pathways, including suppressed FCGR3A phagocytosis and CD209 signaling in B-cells and CD8+ T-cells, as validated via single-cell RNA-seq. Notably, the study uncovered an environmental trait-related signature influencing IL-6 expression via the transcription factor CEBP, distinguishing severe from mild cases and also highlighting the role of neutrophils and CD8+ T-cells as key contributors to interferon-related hypomethylation, providing a molecular basis for the cytokine storm observed in critical COVID-19 [90].

SARS-CoV-2 infection is initiated in the upper respiratory tract, and a study evaluating methylation patterns of nasopharyngeal specimens obtained from hospitalized patients with moderate to severe COVID-19 was performed, considering the differences in the immune cell populations of airway mucosa and blood. Some of the SARS-CoV-2-induced methylation alterations were transient, reverting to normal levels within 6 weeks post-infection. S100 family genes were identified as prognostic markers of severe COVID-19 and the PI3K/Akt intracellular pathway was found to be an important signaling pathway in the cytokine storm induced by SARS-CoV-2 and also in COVID-19 coagulopathies. Notably, the pro-inflammatory factor IL-17A was enriched at inclusion and the antiviral NUP93 factor was enriched at 6 week post-inclusion. RNAse1 and RNAse2 emerged as top regulators, as well as IL-18, a vital factor that influences various biological processes in COVID-19 [91].

An analytical pipeline exploring single-cell DNA methylation variations in COVID-19 patients discovered significant cell-type specificity of methylation profiles. The most enriched with differentially methylated genes cell type was gamma-delta T cells; however, the methylation profiles did not correlate with the severity of the disease. Furthermore, five differentially methylated genes were identified associated with several cell types—*S100A9*, *AHNAK*, *CX3CR1*, *TRAF3IP3* and *LFNG*, associated with the immune and virus-related signaling pathways. The study associated the expression of *CX3CR1*, a member of the chemokine receptor superfamily, mostly expressed on cytotoxic effector lymphocytes (e.g. NK cells, CD8+ T and macrophages), with the pathological mechanism of severe COVID-19, and suggested that the elevated methylation lowered the expression of CX3CR1 in immune cells and contributed to the aberrant antiviral response in COVID-19 [92].

Machine learning algorithms are the prospective tools for the prediction of epimarkers to identify at-risk individuals who would benefit from timely medical interventions. A random forest classification model focusing on methylation status of the four specific CpG sites (*cg10778971*, *cg03753191*, *cg07878065*, *cg17114584*) predicts the disease severity outcome with AUC-ROC of 0.933 and AUC-PRC of 0.965 on the training cohort and AUC-ROC of 0.898 and AUC-PRC of 0.864 on the validation cohort [93].

The main challenge in translating the epigenetic signatures related to the severity of COVID-19, such as those associated with the *AIM2* and *PARP9* genes, is the low reproducibility of the results between studies due to several factors. First, the heterogeneity of the studied populations, since patient cohorts differ in terms of ethnic background, age, and presence of comorbidities. These factors can influence the methylation patterns, making markers specific to a particular population. Second, currently there is a lack of standardization of clinical criteria for the definition of what is concerned “mild” or “severe” COVID-19 cases across studies. This leads to comparisons between different phenotypes, making it challenging to draw meaningful conclusions. Third, the use of different technological platforms and data processing and bioinformatic analysis protocols introduces significant technical noise, making it difficult to compare results. Thus, for clinical implementation, any epigenetic biomarker must undergo rigorous validation in large, prospective, multicenter trials using standardized operational procedures at all stages, from sample collection to data analysis.

### 2.7. Future Directions: Open Questions and Testable Predictions

While the mechanisms linking acute COVID-19 inflammation to long COVID are becoming apparent, several key questions remain unclear, and the answers will guide future research. An essential question is whether an early blockade of IL-6 can prevent the development of long-lived innate immune “scars”, as anti-IL-6 therapy during the acute phase is predicted to suppress the persistence of the open inflammatory enhancers in monocytes and restore normal responsiveness after 3–6 months [24,26]. Another question is the predictive power of evaluation of ciliary methylation levels for the development of respiratory symptoms: hypermethylation and repression of *FOXJ1* and *DNAH5* in nasal mucosa at 4–8 weeks post-infection are expected to forecast the persistence of cough and dyspnea over 3–12 months, building on evidence of sustained ciliary gene repression tied to symptom trajectories [30,31].

An important topic for future investigation is whether the epigenetic aging marks could be reversed after the viral clearance, and if they could be tied to inflammatory peaks. Partial reversibility of the epimarks of age acceleration and correlations with the outcomes were demonstrated, though moderated by cellular composition variations [75,77].

Finally, the potential of small miRNA and v-miRNA panels for minimally invasive diagnostics holds promise: a curated set of 6–10 circulating miRNAs (such as upregulated miR-21-5p and miR-155-5p, alongside downregulated let-7 and miR-29a-3p) plus virion-linked v-miRNAs like CoV2-miR-O7a and O8 is forecasted to stratify infection severity and long COVID risk effectively, validated through plasma qPCR or next-generation sequencing in diverse cohorts. This draws from consistent miRNA dysregulation patterns and the detection of v-miRNA-like RNAs in affected individuals [56,58,59,61,67,68]. These predictions might not only bridge gaps in our understanding of the mechanisms of COVID-19 and the outcomes of the disease, but also unlock the preventive and prognostic tools, transforming long COVID from a diagnostic puzzle into a well-understood, manageable consequence.

## 3. Conclusions

There is a growing body of evidence pointing towards the diverse roles of epigenetic alterations in COVID-19 severity. The pathogenesis of COVID-19 extends far beyond the initial viral infection, revealing a complex and dynamic interplay between SARS-CoV-2 and the host.

The virus affects the body both directly, by modifying signaling cascades, chromatin accessibility, and methylation, and indirectly, by altering immune system function. This is evidenced by the establishment of trained immunity in myeloid progenitors and widespread epigenetic drift across diverse cell populations, from nasal epithelium to peripheral blood cells. These alterations disrupt critical pathways, including ciliary function in the airways, interferon signaling, and coordinated immune activation, thereby dictating disease severity and clinical outcomes. Such epigenetic alterations could be the basis of the phenomenon of long COVID. Epigenetic age acceleration and telomere attrition correlate strongly with disease severity and mortality, suggesting that the viral-induced cytokine storm and cellular senescence lead to premature aging on a physiological level. Furthermore, the persistence of an altered epigenetic landscape in immune and tissue-resident cells weeks and months after the acute infection has resolved offers a mechanistic explanation for chronic symptoms such as fatigue, cognitive dysfunction, and respiratory issues. These findings shift the perspective of COVID-19 from an acute respiratory illness to a systemic condition with deep and lasting molecular imprints.

The identification of highly specific epigenetic signatures, such as the EPICOVID marker set, underscores the potential of epigenetics not only as a powerful predictive biomarker for stratifying patient risk but also paves the way for understanding the molecular basis of the disease and gives promise for the development of new therapeutic approaches. Future research is needed to determine the duration and longitudinal dynamics of epigenetic alterations and to translate these epigenetic insights into effective treatments that can address both the acute and chronic phases of COVID-19.

## Figures and Tables

**Table 1:** Marker genes with altered epigenetic status differentiating severe and mild COVID-19.

Gene Name	Molecular Function	Association with COVID-19 Severity	Methylation Status in Severe COVID-19
*AIM2*	Inflammasome component; involved in interferon response to viral infection	Part of a DNA methylation signature (EPICOVID) associated with clinical severity and respiratory failure.	hypo
*HLA-C*	Crucial determinant of immune function and NK cell activity; involved in interferon response to viral infection.	Part of a DNA methylation signature (EPICOVID) associated with clinical severity and respiratory failure. Methylation levels in men were significantly higher in the severe group.	hypo
*MX1*	Interferon-induced GTP-binding protein; inhibits viral replication by trapping viral components.	Altered DNA methylation profiles are part of a signature with 96.87–100% accuracy in stratifying COVID-19 from other diseases	hypo
*PARP9*	Poly(ADP-ribose) polymerase involved in DNA repair and interferon signaling.	Altered DNA methylation profiles are part of a signature with 96.87–100% accuracy in stratifying COVID-19 from other diseases	hypo
*IRF7*	Involved in interferon signaling and viral response.	Differentially methylated sites were significantly enriched in this gene; methylation signature can predict hospitalization, ICU admission, and progression to death.	hypo
*OAS1*	Involved in interferon signaling and viral response.	Differentially methylated sites were significantly enriched in this gene; methylation signature can predict hospitalization, ICU admission, and progression to death.	hyper

## Data Availability

No new data were created or analyzed in this study. Data sharing is not applicable to this article.
